# The missing link: Predicting connectomes from noisy and partially observed tract tracing data

**DOI:** 10.1371/journal.pcbi.1005374

**Published:** 2017-01-31

**Authors:** Max Hinne, Annet Meijers, Rembrandt Bakker, Paul H. E. Tiesinga, Morten Mørup, Marcel A. J. van Gerven

**Affiliations:** 1 Radboud University, Donders Institute for Brain, Cognition and Behaviour, Nijmegen, the Netherlands; 2 Institute of Neuroscience and Medicine, Institute for Advanced Simulation and JARA BRAIN Institute I, Jülich Research Centre, Jülich, Germany; 3 Technical University of Denmark, DTU Compute, Kgs. Lyngby, Denmark; University of California Berkeley, UNITED STATES

## Abstract

Our understanding of the wiring map of the brain, known as the *connectome*, has increased greatly in the last decade, mostly due to technological advancements in neuroimaging techniques and improvements in computational tools to interpret the vast amount of available data. Despite this, with the exception of the *C. elegans* roundworm, no definitive connectome has been established for any species. In order to obtain this, tracer studies are particularly appealing, as these have proven highly reliable. The downside of tract tracing is that it is costly to perform, and can only be applied *ex vivo*. In this paper, we suggest that instead of probing all possible connections, hitherto unknown connections may be predicted from the data that is already available. Our approach uses a ‘latent space model’ that embeds the connectivity in an abstract physical space. Regions that are close in the latent space have a high chance of being connected, while regions far apart are most likely disconnected in the connectome. After learning the latent embedding from the connections that we did observe, the latent space allows us to predict connections that have not been probed previously. We apply the methodology to two connectivity data sets of the macaque, where we demonstrate that the latent space model is successful in predicting unobserved connectivity, outperforming two baselines and an alternative model in nearly all cases. Furthermore, we show how the latent spatial embedding may be used to integrate multimodal observations (i.e. anterograde and retrograde tracers) for the mouse neocortex. Finally, our probabilistic approach enables us to make explicit which connections are easy to predict and which prove difficult, allowing for informed follow-up studies.

## Author summary

Tract tracing is a highly accurate procedure for identifying animal brain connectivity. However, the technique is labor intensive and requires the sacrifice of animal subjects. In our work, we describe a computational method that is able to predict the presence or absence of unobserved connections, without having to probe these connections physically. The model works by learning for each of the nodes in the connectome its position in a latent space. Nodes that are connected according to the available data are placed close to one another, while disconnected nodes are positioned far apart. Unobserved connections may now be inferred by looking at the corresponding distance in the latent space. We apply the procedure to two data sets of the macaque brain and show that the latent space model is able to predict the strength of unknown connections. Furthermore, we use the model to integrate anterograde and retrograde data for the mouse connectome. Because the model is probabilistic, it allows us to quantify how certain we are about our predictions. This enables future research to determine which connections can confidently predicted, and which connections require further data acquisition.

## Introduction

Recent years have seen a surge in research effort devoted to obtaining the human *connectome*, a map of all the connections in the human brain at the level of macroscopic brain regions [1, 2]. Technological advances, in particular diffusion-weighted MRI (dMRI), have enabled bundles of white-matter fibers to be identified *in vivo* in unprecedented detail. However, dMRI suffers from a number of drawbacks [3–5]. For instance, it is an indirect measuring technique [6]: Rather than directly observing axons or large fiber bundles, these must be inferred from the diffuse movement of water molecules, using a process called tractography. In practice, the problem tractography tries to solve may be underspecified, as a single voxel may contain fibers that cross, ‘kiss’, merge or split [7]. As a result, it may be unclear which path the estimated fibers follow. Further problems arise when interpreting the output of (probabilistic) tractography. The number of streamlines (i.e. candidate fiber trajectories) that connect two regions of interest is often used synonymously with fiber count, yet the actual number of streamlines between two regions is an intricate function of the actual fiber count and several parameters of the dMRI acquisition and tractography procedure [3, 8]. In all, despite dMRI having greatly advanced the field of connectomics by being applicable in living human subjects, it is far from the be-all end-all solution to finding gross anatomical connectivity.

Earlier approaches for studying brain connectivity [9] involve invasive techniques such as post-mortem dissection [10, 11] as well as tract tracing in animal subjects [12]. In the latter approach a tracer (such as a fluorescent dye or a virus) is injected into neuronal tissue of a living animal. After appropriate waiting time, the animal is sacrificed to allow the tracer material to spread through the tissue, either in the direction from cell soma to axon terminal (known as anterograde tracing), or vice versa (retrograde tracing). Inspection of the virus expression or the fluorescence of the dye is subsequently used to determine to which other neuronal populations the injection site was connected [13–15]. Tract tracing has a number of advantages over dMRI-based connectivity estimation. First of all, tract tracing provides unequivocal proof that two regions are connected. In dMRI, there is always a possibility that fiber tracts follow the same highway, but do not mix. Furthermore, tract tracing can recover the direction of the tracts it recovers, something which is impossible to do with dMRI. Furthermore, the probed connections are measured directly, without the need for an additional processing step such as tractography. This results in very accurate connectivity estimates, in particular regarding long-range connections [6, 16] and has prompted researchers to use tract tracing methods as a means to evaluate the performance of dMRI-based structural connectivity estimation [17–19].

Compared to dMRI, tract tracing is a very expensive procedure for probing connectivity [20, 21]. It requires sacrificing animal subjects, as well as substantial manual labor in administering the tracers and processing the treated brain tissue. Through a process known as ‘link prediction’ [22–24] the number of experimental studies needed to evaluate all possible connections in a connectome may be reduced. The general idea behind this technique is that the connections that have been observed carry enough information for the missing connections to be predicted. One class of models used for making link predictions assumes that connections are the result of hidden properties of the nodes in the network (i.e. regions of interest or neuronal populations) [25]. For instance, stochastic block models assume the nodes of a network have latent class labels, and that the probability of a connection between two nodes depends on whether they share the same label [26]. By learning this latent structure from the data, i.e. which node has which label, new connections (or the absence thereof) may be predicted [27–31]. The concept of latent node classes also forms the basis of community detection [32], for which the goal is to identify sets of nodes that have more connections among themselves than with nodes outside the set. Another latent structure approach assumes that the nodes of a network are actually embedded in an unknown physical space (a ‘latent space’) [33, 34]. When a latent space model (LSM) is used for link prediction, the (Euclidean) distance between the positions of nodes in the latent space is used to determine the likelihood of a connection. This approach is clearly applicable when networks represent geographically restricted phenomena, like traffic, power grids and the internet, but may also be used in more abstract settings, such as a social network with ties dependent on political ideology, rather than spatial location [33].

While stochastic block models have been used for modeling and prediction of links in structural connectivity [35–37], LSMs have so far mostly been applied to social network analysis [38–40] instead of to structural connectivity. However, clearly the connectome is spatially embedded [41–43], suggesting that the use of LSM can improve the quality of link prediction. In the current study, we describe an extended probabilistic LSM with which we embed tract-tracing connectomes into a latent space. This allows us to predict unknown connections in macaque visual cortex [44] and macaque cerebral cortex [45]. Additionally, the procedure is applied to combine anterograde and retrograde tract tracing data for the mouse neocortex [46]. While in this data set all connections have been observed, the different tracer directions disagree about connection strengths. We show that by embedding the network into a latent space, both sources of data can be explained by a single connectome.

The predictive performance of the LSM is compared with two baseline models as well as with the more general latent eigenmodel, as described in [25]. Our analyses demonstrate that the LSM clearly outperforms the baseline model and slightly improves on the latent eigenmodel. The probabilistic nature of our approach provides an intuitive representation of the uncertainty in the parameters we estimate, in the form of their posterior distribution. This uncertainty may be used to determine which predicted connections are reliable and which require more data to be estimated with confidence. Finally, the spatial embedding obtained by the LSM may be interpreted to gain additional insight in the structural organization of a connectome.

## Materials and methods

### Data

The data sets used in this paper are publicly available. Surface data was available for the macaque data sets, but not for the mouse data as the node definitions for this data set are layer-specific. The properties of each of the data sets are summarized in Table 1 and discussed in detail below.

**Table 1:** For each of the different connectivity data sets, the table shows the number of source nodes, the number of target nodes, the numbers of observed and unobserved connections and finally the number of observed connection strength classes *K*.

Connectome	Sources	Targets	Observed	Unobserved	*K*
Macaque visual system	32	32	653	339	2
Macaque cerebral cortex	91	29	2610	5580	4
Mouse neocortex, anterograde only	49	49	2352	—	4
Mouse neocortex, retrograde only	49	49	2352	—	4
Mouse neocortex, both modalities	49	49	2352	—	4

#### Macaque visual system

The macaque visual system connectome consists of the combined results of 31 studies, collected by [44]. The result is a partially observed connectome of size 32 × 32, consisting of both anterograde and retrograde tracings for one hemisphere. Connections are classified as either absent, present or unknown. Of the 32 ⋅ 31 = 992 possible connections, 653 candidate connections have been probed and of these, 286 are considered to represent connected node pairs. The other 339 connections remain unknown, and will be predicted using the proposed method.

#### Macaque cerebral cortex

A macaque cerebral cortex connectome was obtained by [45] by injecting retrograde tracers into 29 of 91 architectonic areas, all mapped to the left hemisphere. The result is a partially observed connectome of size 91 × 29. Connection strengths are quantified using the extrinsic fraction of labeled neurons (FLNe) index, which is the fraction of labeled neurons in the source area (i.e. those that send a projection to the injection site), divided by the total number of labeled neurons in the brain except for those in the injection area. Although these scores provide a continuous scale, [45] propose a set of thresholds to categorize the connections into strong, moderate, sparse and absent. Throughout this paper, we use this ordinal representation to predict the unobserved connections.

#### Mouse neocortex

**Fig 1:**
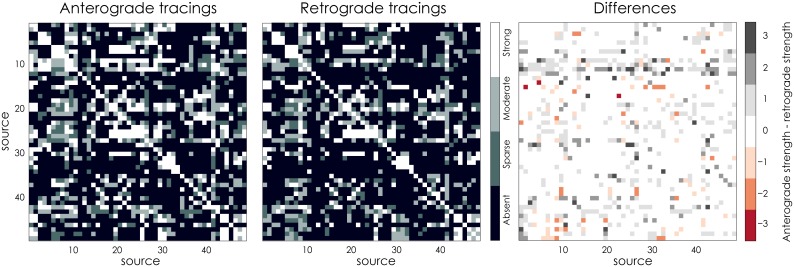
The mouse neocortex data. [46] From left to right: anterograde traced connections, retrograde traced connections and the differences in connections strength between the two modalities. To compute the latter, the connection strengths were represented numerically as absent = 0, sparse = 1, moderate = 2 and strong = 3.

[46] have collected both anterograde and retrograde tracings for the mouse neocortex, which have been aggregated into two 49 × 49 connectivity matrices, shown in Fig 1A and 1B. The connection strengths are labelled strong, moderate, sparse or absent. While the connectomes have been fully observed and therefore contain no missing connections, the anterograde and retrograde tracings are not in complete agreement, as is shown in Fig 1C. This may for example be due to experimental variability, e.g. differences in volume or location of injections, or different sensitivity for the retrograde or anterograde tracers. It is unclear how the two data sources may best be combined. In [46], the combination is performed using a logical AND-operator on the two matrices: a connection is considered present if it is present in both observations. The result is a binary connectome, in which the information contained in the connection strengths is effectively lost. In the following, we will use our methodology to estimate a single connectome using both sources of data, thus cleaning up and reconciling the experimental variability.

### The latent space model

The goal of our method is to predict connectivity for potential connections for which no tracer data is available, informed by the connections that do have observed data. To accomplish this, we assume that the *p* nodes of the network are in fact embedded in a latent space with dimensionality *D*, so that each node *i* has a latent position $z_{i}\in {\text{R}}^{D}$ [33, 34, 38, 39, 41]. Furthermore, we assume that the propensity for two nodes to be connected, or the strength of such a connection, is inversely proportional to the distance *l*_*ij*_ = ‖**z**_*i*_ − **z**_*j*_‖_2_ between the two nodes in the latent space. If no tracer data was available, the nodes are considered to be distributed uniformly within this latent space. As soon as connections between pairs of nodes become observed, this latent arrangement becomes constrained—for example, nodes that are strongly connected should be close to each other and conversely, disconnected nodes should be far apart. The higher the dimensionality of the latent space, the more complex configurations of the connectome the model can represent. For example, in a 1D model the latent positions are ordered on a line, which can host only a limited number of different connectivity structures. On the other hand, in a high-dimensional space the degrees of freedom of the model will be sufficiently high to capture a more complex network topology (although for our purposes, a high-dimensional latent space will be prone to overfitting).

As tracer data is typically available in a thresholded form, e.g. binary connections or ordinal connection weights, the latent space is accompanied by a set of boundaries that determine which range of distances corresponds to a particular connection weight. This idea is implemented using an ordinal regression model [25, 47]. It defines the probability of an ordinal connection class *k* between nodes *i* and *j* as *f*_*ijk*_ = Φ(*i*, *j*, *k*) − Φ(*i*, *j*, *k* − 1), in which

$$\Phi \left(i,j,k\right)=\int_{-\infty }^{h(i,j,k)}N\left(x|0,1\right)\;dx$$ (1)

gives the cumulative density of the standard normal distribution on the interval [−∞, *h*(*i*, *j*, *k*)]. Here, *h*(*i*, *j*, *k*) = (*b*_*k*_ − *l*_*ij*_)/*σ* serves to scale and translate the Euclidean distance *l*_*ij*_ in the latent space to the intervals of the standard normal density function. Note that *b*_*k*_ and *σ* are the same across all connections. The observed connection weights **A** = {*a*_*ij*_} are assumed to follow a categorical distribution with probability vector **f**_*ij*_, subject to 0 ≤ *f*_*ijk*_ ≤ 1 and ∑_*k*_ *f*_*ijk*_ = 1. If anterograde and retrograde tracer data are available separately, as in the mouse data collected by [46], both **A** = {*a*_*ij*_} and **R** = {*r*_*ij*_} follow such a distribution.

Importantly, once the latent positions **z**_*i*_ and the class boundaries *h*_*ijk*_ have been learned using the available observations, the same parameters can be used to predict the class weight probabilities **f**_*ij*_ for unobserved connections, and subsequently predict the values for the missing observations in **A**. Thus, the latent space model as described here serves as a mechanism to ‘complete’ a partially observed connectome.

The latent space model describes only symmetric connectivity behavior, as the Euclidean distance is a symmetric function. However, some part of the connectome topology may not be explained by Euclidean distance alone. For example, some nodes may be hubs—nodes which have a large number of connections compared to the rest. To allow this phenomenon in our model, we add two random vectors that model the additional likelihood of nodes having incoming and outgoing connections, using the vectors $\delta \in {\text{R}}^{p}$ and $\varepsilon \in {\text{R}}^{p}$, respectively [48]. When using these additional factors, we write instead *h*(*i*, *j*, *k*) = (*b*_*k*_ − *l*_*ij*_ + *δ*_*i*_ + *ε*_*j*_)/*σ*. The generative model complete with hyperpriors is described in detail in S1 Appendix, which also discusses constraints to make the model identifiable. Importantly, connections for which no tracer data is observed, provide no information to the model. Instead, by finding the spatial embedding of nodes within the latent space, the ordinal probabilities **f**_*ij*_ for these unknown connections may be inferred. The result is a probabilistic description of a connectome that assigns for each connection a probability for each ordinal category.

To compute the posterior distribution of the latent locations and connection class probabilities, a Hamiltonian Monte Carlo sampling scheme is used [49], which was implemented in Stan [50]. For more detail we refer the reader to S1 Appendix. The result of this procedure is a collection of samples that collectively represent the distribution over the parameters of interest, i.e. the latent positions and the unobserved connection weights.

### Optimal number of dimensions

To determine the optimal dimensionality of the latent space, 10-fold cross-validation was used for each of the different data sets. For each fold, the model was trained using nine-tenth of the observed connections and evaluated using the likelihood of the remaining one-tenth. To evaluate the performance of different numbers of dimensions, the parameter *D* was varied in the range [1, …, 5]. The dimensionality $\hat{D}$ that resulted in the best generalizability (i.e. highest likelihood on the withheld data) was considered the optimal dimensionality. The model was then trained using $\hat{D}$ and all available data.

### Performance measures

The performance of the predicted connectivity is evaluated using the cross-validation results. Per fold, we first compute for the *t*th collected sample $A^{\left(t\right)}=\left\{a_{ij}^{\left(t\right)}\right\}$ the absolute difference between the predicted connection weights $a_{ij}^{\left(t\right)}$ and the observed connection weight *a*_*ij*_, which is subsequently averaged over all connections, i.e.

$$e_{\text{abs}}=\frac{1}{\left|F\right|}\sum_{(i,j)\in F}\left|a_{ij}^{\left(t\right)}-a_{ij}\right|\;,$$ (2)

in which F is the set of edges (*i*, *j*) in that particular cross-validation fold. This error measure is in the range [0, *K* − 1]. Note that *e*_abs_ is a conservative measure of the performance, as Bayesian averaging would typically reduce the error. However, to be consistent with the error measures described next, we evaluate *e*_abs_ per sample.

Depending on the intended application of the predictions, it may be more relevant to consider only the presence or absence of a connection instead of the difference in connection weight. In other words, predicting a weak connection that should have been absent may be a more severe error than predicting a strong connection that is in fact only moderate. We therefore also compute the false positive rate *e*_fpr_ and false negative rate *e*_fnr_, as

$$e_{\text{fpr}}=\frac{FP}{FP+TN}\;e_{\text{fnr}}=\frac{FN}{FN+TP}\;,$$ (3)

with the terms given by $FP=\sum_{(i,j)\in F}1\left[a_{ij}^{\left(t\right)}>0\wedge a_{ij}=0\right]$, $TN=\sum_{(i,j)\in F}1\left[a_{ij}=0\right]$, $FN=\sum_{(i,j)\in F}1\left[a_{ij}^{\left(t\right)}=0\wedge a_{ij}>0\right]$ and $TP=\sum_{(i,j)\in F}1\left[a_{ij}>0\right]$. For each error measure, the presented results are subsequently averaged over all samples and over the cross-validation folds.

In addition to the prediction error, the probabilistic approach to latent space models allows us to compute the uncertainty that is associated with the predictions. To do so, we consider the posterior distributions over the parameters of interest. The width of these distributions provides a measure of uncertainty. If the model is certain about a prediction, the posterior will be peaked around the mode of the distribution, but if there is a lot of uncertainty the distribution will be more dispersed. To analyze how certain the predictions are, we quantify for each of the estimated parameters *f*_*ijk*_ the associated uncertainty as the 95% credible interval, i.e. the width of the range in which 95% of the posterior probability density lies. For each connection between node pairs (*i*, *j*), the largest uncertainty of the *K* possible connection strength classes is reported as the final measure of uncertainty.

### Alternative prediction models

Two baselines were constructed in order to interpret the performance of the latent space model. In the first, connection probabilities for a particular connection strength class *k* are determined by the fraction of connections in the training data having connection weight *k*. In this baseline, the probability vector **f**_*ij*_ is the same for all pairs (*i*, *j*). In the second baseline, a zero-dimensional latent space is used. In other words, the only flexibility the model has, is in the random effects ***δ*** and ***ε***. This baseline serves to evaluate the additional effect of the latent space, compared to the predictive performance of the degree distribution of the training data.

To compare with a more flexible model, we use the latent eigenmodel that was introduced by [25]. Here, the distance *l*_*ij*_ = ‖**z**_*i*_ − **z**_*j*_‖_2_ is replaced by $l_{ij}=-z_{i}^{T}\Lambda z_{j}$, with $\Lambda \in {\text{R}}^{D\times D}$ a diagonal matrix. The elements *λ*_*ii*_ may be interpreted as the relative weights of each of the latent node attributes, which no longer (necessarily) represent spatial position. As described in [25], the latent eigenmodel may serve as a weak generalization of a latent distance model by setting **Λ** to identity. The baselines and latent eigenmodel are described in detail in S1 Appendix.

For the two macaque data sets their respective cortical surfaces were available. This allowed us to determine the position of each region of interest as the center-of-gravity of the vertices in that region. This was used for what we will refer to as a fixed-position model, in which all parameters of the latent space model are learned as before, but the latent positions **z** are determined by the surface. For the mouse data, we were unable to find a cortical reconstruction for the parcellation used in [46], and hence the corresponding analyses have been omitted for this data set.

### Visualization of the latent space

Visualizing the latent space embedding is not straightforward, as the different samples of the posterior may be rotated, translated and scaled relative to each other. Furthermore, the latent space is potentially high-dimensional. An approximate solution to these issues is to use (classical) multidimensional scaling, which visualizes the node positions in an arbitrary number of dimensions, while minimizing the error with respect to the relative distances between the nodes [51]. Throughout the remainder of this paper, we show both the anatomical positioning of nodes on the cortex, as well as the latent space embedding, on a two-dimensional plane, using this procedure. For the latent embedding, we use the posterior expectation of the distances $\hat{L}=\left\{{\hat{l}}_{ij}\right\}$, with ${\hat{l}}_{ij}=\frac{1}{T}\sum_{t=1}^{T}l_{ij}^{\left(t\right)}$, with *T* the number of samples. For the connections, we use the posterior expectation of the connectomes $\hat{A}=\left\{{\hat{a}}_{ij}\right\}$, with ${\hat{a}}_{ij}=\frac{1}{T}\sum_{t=1}^{T}a_{ij}^{\left(t\right)}$. Subsequent to multidimensional scaling, the latent embeddings are transformed using the Procrustean transform [38], using the empirical positions as reference. This approach keeps the relative distances between the nodes intact, but rotates the system so that it is maximally similar to the reference. Note that this step is only applied when a reference is available, i.e. for the two macaque data sets.

## Results

**Fig 2:**
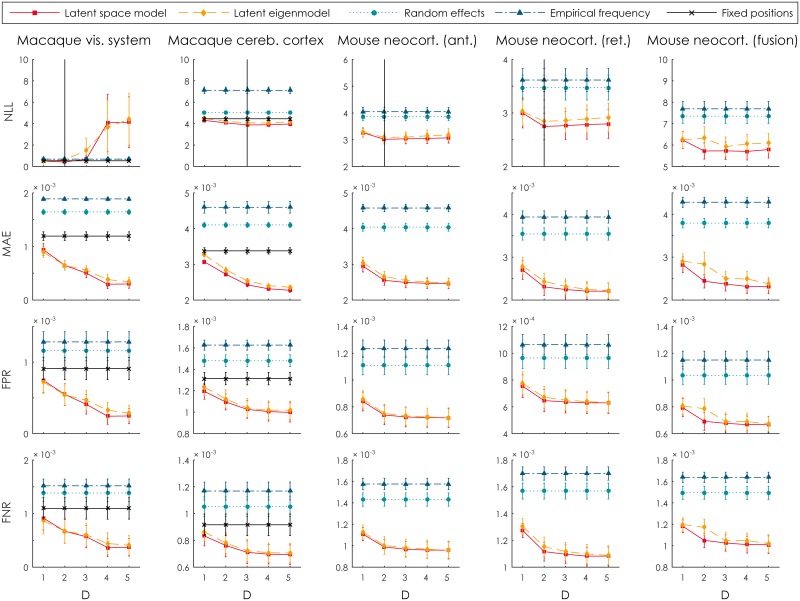
Model performance. The prediction performance of the latent space model, the latent eigenmodel and the two baseline approaches, quantified using the negative log-likelihood (NLL), the mean absolute error (MAE), the false-positive rate (FPR) and the false-negative rate (FNR). All measures are obtained using ten-fold cross-validation. Error bars indicate one standard deviation over the ten folds. In the top row, the number of dimensions $\hat{D}$ with the best generalization performance (i.e. the highest likelihood on hold-out data) is indicated with a vertical line. For the two macaque data sets, results are also shown for the fixed-positions model (see Section Link prediction). All scores have been normalized by the number of testing connections, for comparison between the different data sets.

The latent space model, the latent eigenmodel and the two baseline models were applied to each of the data sets described in Section Data. For the macaque data sets, the fixed-point model was applied as well. Using cross-validation, we computed the (negative) log-likelihood, the mean absolute error as well as false-positive and false-negative rates on the held-out connections. The results are shown in Fig 2. The figure also indicates the dimensionality $\hat{D}$ with the highest generalization performance in terms of model likelihood. The LSM consistently outperforms all of the alternatives when using the dimensionality determined by cross-validation, and in most cases also when using alternative numbers of dimensions. For the macaque visual system, mouse neocortex (anterograde tracers) and mouse neocortex (retrograde tracers), two-dimensional latent space appears to be optimal. For the macaque cerebral cortex, three latent dimensions resulted in the highest likelihood on unseen connections. When combining anterograde and retrograde tracers for the mouse neocortex, four latent dimensions resulted in the best performance, although the difference between two, three or four latent dimensions is minor. In the remainder of our analyses, unless otherwise indicated, we focus on the latent space model as it provides the best predictive performance and has an intuitive interpretation. The predicted connectomes for the dimensionalities other than $\hat{D}$ are show in S1 Appendix. The supplementary material also contains a breakdown of the performance measures into connections that have been observed in both directions, and those connections for which only the anterograde or retrograde observation is available. Note that this only applies to the two macaque data sets, as the mouse neocortex data is fully observed.

Interestingly, as Fig 2 shows, while the log-likelihood shows an optimum at the indicated numbers of dimensions, the other error measures continue to improve as *D* increases. This is due to the log-likelihood being the only measure that explicitly takes the connection probabilities **f**_*ij*_ (and thus, indirectly, the latent positions) into account, instead of considering the resulting connections *a*_*ij*_.

By supplying the model with the actual positions on the cortex, we learn the amount of variability that can be explained by the physical distances alone. Fig 2 shows that of the three baseline models, this approach has by far the best predictive performance. For the macaque visual system, the fixed-positions baseline is slightly improved upon by the LSM for dimensionality 1 and 2. For the macaque cerebral cortex the additional benefit of the LSM is substantially larger, but here too the anatomical positions outperform the other two baselines.

**Fig 3:**
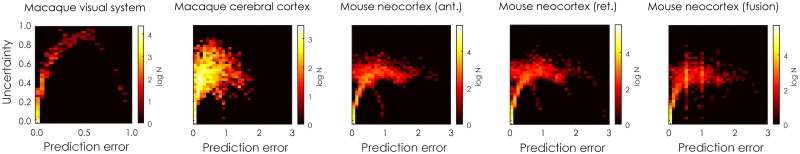
Prediction error and uncertainty. The relationship between prediction error and uncertainty, by the LSM. Colors are determined by the number of connections that lie within each cell; warmer colors indicate more connections. Counts are log-transformed for visualization. Note that for the mouse neocortex data fusion case, the prediction error is averaged over errors with the anterograde and retrograde data.

In addition to the performance measures, we used the cross-validation approach to compute the uncertainty that is associated with each of the estimated connections, and plotted these as a function of the prediction error, as shown in Fig 3. For each data set, the optimal dimensionality $\hat{D}$ was used as indicated in Fig 2. The diagrams show that lower uncertainty typically goes hand-in-hand with low prediction errors, and vice versa. This implies that in absence of a ground truth, the prediction uncertainty may be used as a proxy for prediction quality. In other words, connections about which the model is certain tend to be estimated correctly.

**Fig 4:**
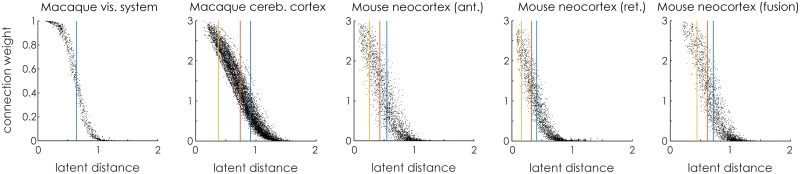
Latent distances versus connection weights. For each connection, the latent distance is shown as well as the posterior expectation of the connection weight. The expectations of the *K* − 1 boundaries between the difference connection weight classes *b*_*k*_ are indicated with vertical lines.

**Fig 5:**
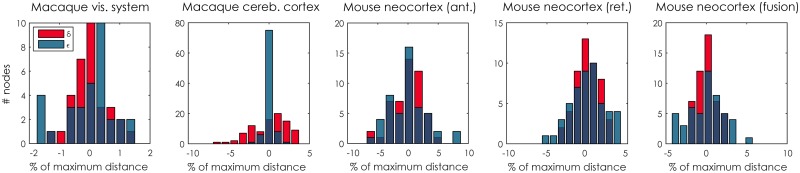
Magnitudes of source and target effects. Connections weights may be modulated properties of their end points, modeled here as source ***δ*** and target ***ε*** effects. A positive effect *δ*_*i*_ or *ε*_*i*_ means an increased likelihood of a connection originating from or terminating at node *i*, respectively. The effects have been scaled to percentages of the maximum latent distance ($2\sqrt{\hat{D}}$) for easier interpretation.

With the optimal dimensionality determined as above, we trained the LSM on all observed connections. To illustrate the parameters that the model learns, Fig 4 shows the posterior expectation of the connection weight for each connection, as a function of the latent distance of that connection. Superimposed are the expectations of the *K* − 1 boundaries *b*_*k*_ that indicate the latent distances at which the model transitions from predicting a particular connection weight to another. Note that connections with the same latent distance may still be assigned a different weight, due to the random effects ***δ*** and ***ε***. The magnitude of these effects is shown in Fig 5. The histograms show that the effects of the source and target effects is relatively small, corresponding to at most a latent distance of 10% of the maximum distance possible. For the macaque cerebral cortex data set, many nodes (75 of 91) have *ε* = 0, which is caused by this data set having only 29 out of the 91 regions with retrograde connections observed.

In the remainder of this section, we first consider the prediction of unobserved connectivity for the two macaque data sets, and second describe the data fusion approach for the mouse neocortex data.

### Link prediction

#### Macaque visual system

**Fig 6:**
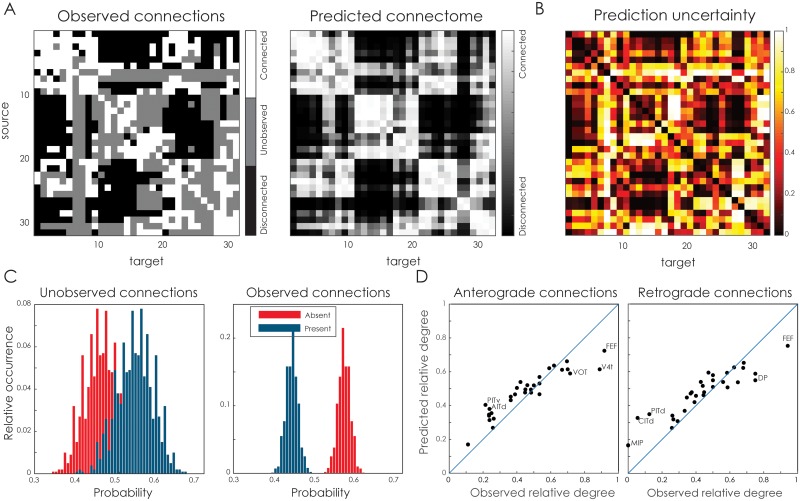
Macaque visual system connectivity. **A**. The observed tracing data (left) [44], the corresponding predicted connectome (right), based on the 2D latent space model. **B**. The uncertainty associated with each of the predicted connections, indicated by the width of the 95% credible interval for the most uncertain class (see text). **C**. The predicted fraction of absent and present edges for either unobserved connections (left panel) or observed connections (right panel). **D**. The observed versus the predicted relative degree of the nodes in the network, for anterograde connections (left panel) and retrograde connections (right panel). The top five nodes with the largest differences in relative degree have their labels shown. A full listing is provided in S1 Appendix.

Fig 6 shows the result for predicted connectivity for the macaque visual cortex. Fig 6A shows the original observations, in which a number of connections are marked as unknown, together with the posterior expectation (i.e. the mean of the posterior samples) of the predicted connectivity, using a two-dimensional latent space. For each connection, the associated uncertainty is shown in Fig 6B. Naturally, the connections with the highest uncertainty are the unobserved ones (see also Fig 3). A decent number of connections have a low uncertainty (32% of the connections have an uncertainty below 0.2; 57% have an uncertainty below 0.5), demonstrating that the LSM is able to conform to many of the observed connections. In Fig 6C, the probabilities of each connection strength class are shown separately for the unobserved and observed connections. Note that because the macaque visual connectome is binary, the figure contains some redundancy as the probability of a present connection is simply one minus the probability of an absent connection; hence the histograms are mirror images of each other. The histograms in the left panel show that approximately 55% of the unknown connections are predicted to exist. The model does not adopt the same distribution of present edges as in the observed connections, as can be seen from the differences between the two panels. This further corroborates that useful information is captured in the latent node positioning and that the empirical frequency baseline is insufficient to predict connections (cf. Fig 2).

To understand how connectivity is organized according to the latent space model predictions, we first compute for each node its relative degree i.e. number of connections of a node divided by the number of possible connections, using either only the empirically observed connections or the completed connectome using the LSM. Fig 6D shows a scatter plot of these relative degrees, which indicates that the predicted connections are not distributed homogeneously over the connectome, but that some regions have more newly predicted connections than others. For example, the frontal eye field (FEF) becomes relatively less connected, while the posterior inferotemporal area (PITv and PITd) have more connections in the predicted connectome than in the observations alone. A full listing of the relative degrees of each node is shown in S1 Appendix.

**Fig 7:**
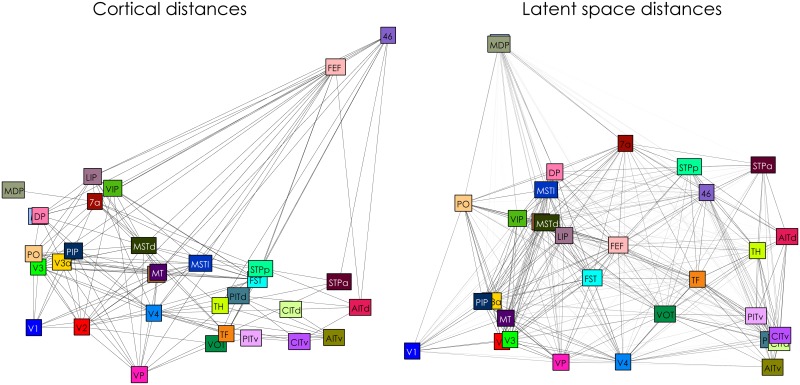
Macaque visual system positioning. The left panel shows the node positions determined by the physical distances between the ROI. The connectivity in the left panel consists of the observed and present connections in the data. The right panel shows the posterior expectations of node distances as determined by the latent space model, with optimal dimensionality $\hat{D}=2$. Note positions are approximated using multidimensional scaling, based on the distances on the cortex and the posterior expectation of distances in the latent space.

By visualizing the latent embedding, this behaviour becomes more apparent. Fig 7 shows both the observed connections and positions (note that observed absent connections and unknown connections both appear as the absence of an edge) and the posterior expectations of the latent embedding and connectivity. The predicted connectome preserves the general anatomical structure of the cortex, as can be seen from the clustering of early visual areas, as well as of the inferotemporal and parietal regions. Despite these similarities, there are also a number of nodes that are placed substantially differently in the latent embedding. For example, prefrontal area 46 and the frontal eye field are located centrally in the predicted connectome, due to their strong connectivity to other regions, while on the cortex these areas are obviously in frontal cortex. In contrast, medial parietal areas MIP and MDP are moved towards the outskirts of the connectome, as they are predicted to connect only sparsely to the other regions (see also their respective rows in Fig 6A). Overall, the correlation between the cortical distances and those of the posterior is 0.45 (SD = 0.03).

#### Macaque cerebral cortex

**Fig 8:**
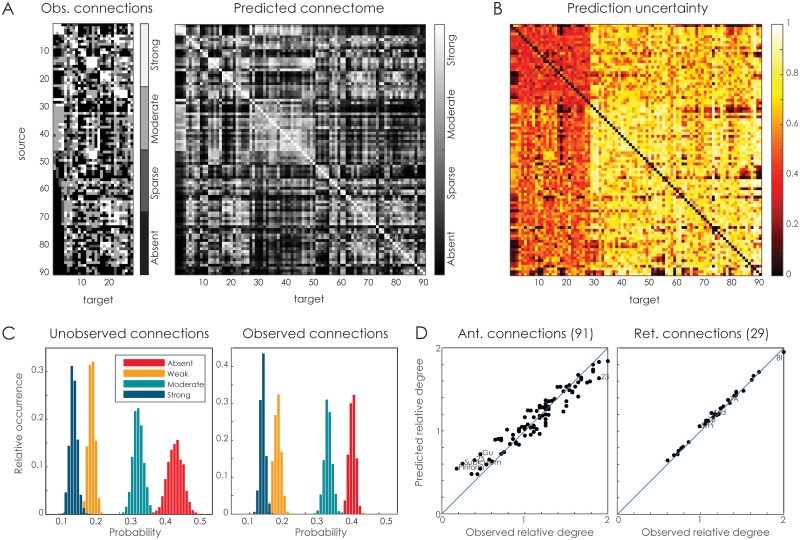
Macaque cerebral cortex connectivity. **A**. The observed tracing data (left) [45], the corresponding predicted connectome (right), based on the 3D latent space model. **B**. The uncertainty associated with each of the predicted connections. **C**. The predicted fraction of each of the connection weight classes for either unobserved connections (left panel) or observed connections (right panel). **D**. The observed versus the predicted relative degree of the nodes in the network, for anterograde connections (left panel, 91 nodes) and retrograde connections (right panel, 29 nodes). The top five nodes with the largest differences in relative degree have their labels shown. A full listing is provided in S1 Appendix.

Similarly, Fig 8 shows the prediction results for the macaque cerebral cortex. Here the lack of observations is systematic, as only the first 29 regions have been injected with a retrograde tracer. Fig 8A shows the observed connections based on these injections as well as the posterior expectation of connectivity using the latent space model with three latent dimensions. The associated prediction uncertainty is shown in Fig 8B. Here, the most certain estimates are obviously obtained for the off-diagonal elements of the 29 × 29 submatrix for which connectivity in both directions is observed (note that the diagonal, i.e. self-connections, are not predicted). The certainty is lower for the off-diagonal elements of the (91 − 29) × 29 submatrix for which retrograde connections are observed (but no anterograde connections). The remaining 91 × (91 − 29) matrix contains connections that have not been observed at all and are predicted solely on the learned latent positions. Here, the uncertainty is the highest, although a number of connections may be predicted with confidence nonetheless. Fig 8C shows the distributions of connection weight classes over the collected samples, for either the unobserved connections or the observed connections. The distributions differ slightly; in particular the ‘absent’ class has a broader distribution for the unobserved connections. Finally, Fig 8D shows for the 91 regions with observed outgoing connections (left panel) and for the injected 29 regions with incoming connections (right panel) the difference in connections per node. Since for the 29 injection sites connectivity is fully observed in both directions, the model fits closely to the empirical data and does not deviate substantially in terms of node degree. For the 91 target sites, some differences can be identified. For example, the piriform cortex becomes more strongly connected to the network in the predicted connectome, whereas area 23 (in the posterior cingulate gyrus) becomes connected less strongly. A full listing of the predicted and observed degrees is provided in S1 Appendix.

**Fig 9:**
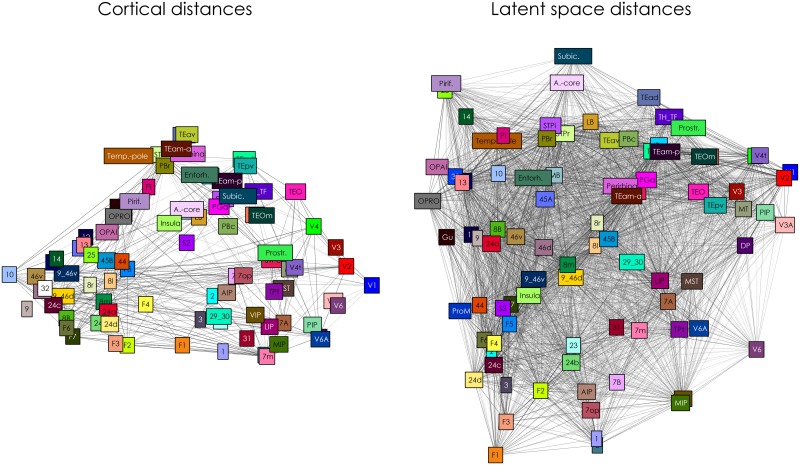
Macaque cerebral cortex positioning. The left panel shows the node positions determined by the physical distances of the ROI. The connectivity in the left panel consists of the observed and present connections in the data. The right panel shows the posterior expectations of node distances as determined by the latent space model, with optimal dimensionality $\hat{D}=3$. Note positions are approximated using multidimensional scaling, based on the distances on the cortex and the posterior expectation of distances in the latent space.

Fig 9 shows the physical as well as the latent positions of the nodes in the macaque cerebral cortex connectome. The latent embedding appears to follow gross anatomical constraints and places similar regions close to each other, e.g. early visual cortex, temporal cortex, frontal cortex. Some regions are placed more peripherally than on the actual cortex. For example, visuomotor areas V6a and V6 are placed further away from later visual areas such as V4, while on the cortex these regions are all closely knit together. This is a result of the distinct connectivity profile for these areas, that makes V6 and V6a more different from early visual areas than their anatomical location would lead to expect (see also the corresponding rows of Fig 8A for the connectivity vectors of these areas). A number of other differences can be observed, such as the boundary positions of the auditory core and the subiculum. The correlation between the cortical and posterior distances is 0.47 (SD = 0.02).

### Integrating anterograde and retrograde data

Instead of predicting unobserved connections, the latent space model may also be used to integrate different modalities. Here, we combine both anterograde and retrograde tracing data collected for the mouse neocortex [46] into a unifying estimate of the underlying connectome. The model captures the remaining asymmetry using the random effects parameters ***δ*** and ***ε***. As shown in Fig 2, the best generalization performance is obtained using a two-dimensional latent space when using either retrograde or anterograde tracers on their own. However, once the data are combined, a four-dimensional space is optimal instead.

**Fig 10:**
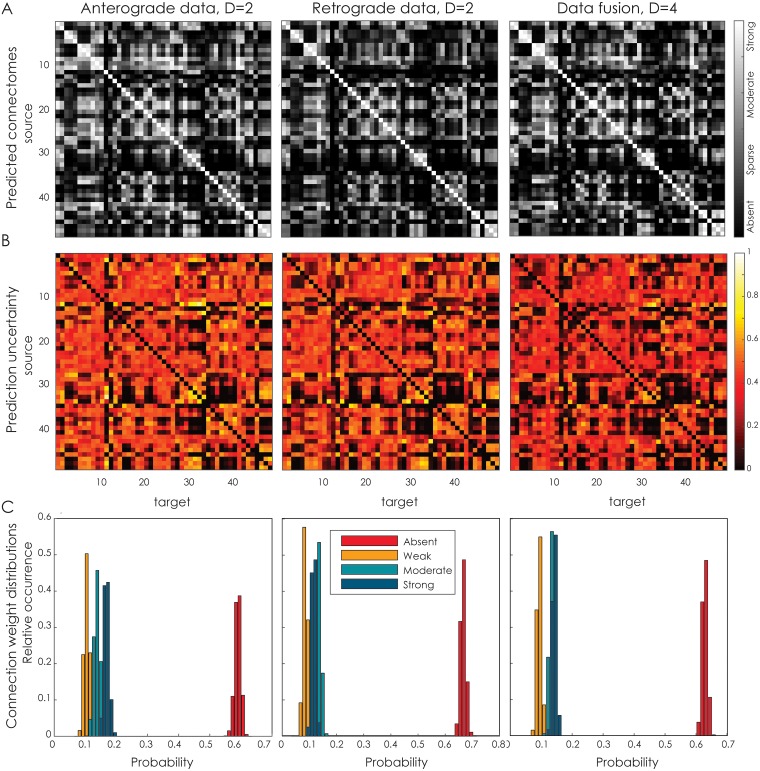
Combined mouse neocortex connectivity. **A**. Posterior expectation of connectivity for the mouse neocortex [46], using either anterograde data only (using two latent dimensions), retrograde data only (using two latent dimensions), or the combination of both (using four latent dimensions, see main text). **B**. The uncertainty associated with these predictions. **C**. The distribution of connection weights for the three predictions.

Fig 10A shows the predicted connectome for the mouse neocortex using either a single data source with a latent space dimensionality of two, or the combined data sources with a latent space dimensionality of four. The overall structure of the matrices appears to be the same in either setting. In Fig 10, the uncertainty that comes with the predictions is shown. The average uncertainty when using only anterograde data is 0.35 (SD = 0.20), for only retrograde data 0.32 (SD = 0.20) and 0.29 (SD = 0.19) when the modalities are combined, demonstrating that the resulting connectome is predicted with slightly more confidence when both modalities are used simultaneously. The histograms in Fig 10C show that the distribution of connection weights remains approximately the same for the three conditions, but are most peaked for the data fusion case.

**Fig 11:**
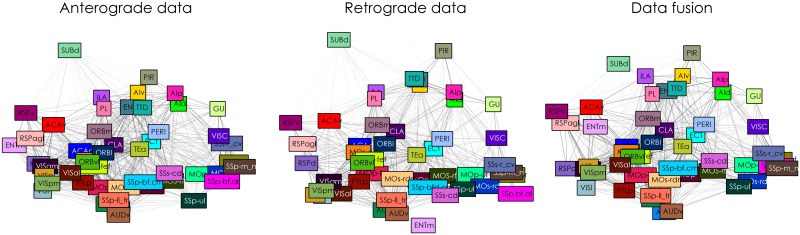
Mouse neocortex connectivity. Posterior expectation of node distances and connectivity using either only anterograde (left panel), retrograde (middle panel), or both (right panel) tracer data. Node distances are determined using a two-dimensional latent space for the single-modality cases and a four-dimensional latent space. Node positions are approximated using multidimensional scaling.

In Fig 11, the latent embedding is shown for the two single-modality connectomes, as well as for the data fusion approach. The most clear example of a region that is connected differently according to the single-modality connectomes is the medial entorhinal cortex (ENTm). It has outgoing connectivity to retrosplenial areas (RSP) and visual cortex (VIS), but receives only a few connections itself, mainly from olfactory areas such as PIR and insular cortex (GU). This causes it to be placed nearly disconnected in the retrograde tracer case. When both modalities are used, ENTm is placed closer to retrosplenial areas as it is in the anterograde tracer connectome.

## Discussion

We have demonstrated that connectivity can be predicted between regions for which no data has been observed directly. We accomplished this by estimating a latent space in which the nodes of the connectome are embedded. In this latent space, the distances between nodes determine the probability or strength of the corresponding connections: nodes that are close together are more likely to be connected (or have a higher connection strength) than nodes that are far apart. The latent space model (LSM) was applied to predict connectivity for two connectomes of the macaque, as well as to integrate anterograde and retrograde tracer data for the mouse neocortex. We evaluated the prediction performance by comparing the LSM with two baselines and a more general (hence less constrained) model known as the latent eigenmodel (LEM) [25]. Our results indicate that the LSM predictions were accompanied by low average errors and typically outperformed the baselines and the LEM. Although the LEM is a more general latent variable, the Euclidean distance function of the LSM appears beneficial for capturing connectivity structure.

By visualizing the latent embedding of nodes in comparison to their positions on the actual cortex (available for the two macaque data sets), it becomes apparent which regions have ‘surprising’ locations if we consider solely their connectivity. For the macaque visual system connectome for example, the frontal eye field and prefrontal area 46 are strongly connected to other visual areas, which causes the LSM to place them more closely to these areas than they are on the cortex. For the cerebral cortex connectome we observe the opposite; regions V6 and V6a are placed further away from early visual cortex than in actual anatomy. The fact that the number of regions that are located surprisingly in this sense is small, indicates further that connectivity and physical distance often go hand in hand.

When many of the connections are actually not observed (as for the macaque cerebral cortex connectome), the model may be uncertain about its predictions. As the described LSM is probabilistic, this prediction uncertainty can be made explicit. This revealed that even though on average the model performs well, depending on the data a substantial number of (potential) connections could not be predicted with confidence. This is due to too few data points being available for the involved regions, so that the LSM cannot fixate their positions in the latent space. The representation of uncertainty provides target areas for additional experiments with novel tracers. Those connections with maximal uncertainty should be probed first in future work, so that these connections become known, and the latent space parameters with the most degrees of freedom become anchored, which will propagate to making other predictions more certain as well. Our approach is therefore applicable both for prediction of unseen connections, as well as for guidance of optimal experimental design [52, 53].

The prediction performance and certainty may be increased by using alternative statistical models for prediction, such as a non-parametric Gaussian process based approach [54], or different latent variable models. However, in the current paper and the context of connectomics, the choice for a latent space model was motivated by findings that show that the probability of two regions being connected (and the strength of this connection) is inversely correlated with the Euclidean distance between them [42, 43, 55, 56]. This made it likely that a LSM could fit well to the data and has the additional benefit of an interpretable representation, which more advanced statistical procedures may lack. We observed that this was indeed the case, and that only a small number of latent dimensions sufficed in modelling each of the considered connectomes. The results of the fixed-positions baseline (in which the latent positions were replaced by the anatomical centers-of-gravity of the regions involved) further corroborate that indeed much of the connectome can be explained by the physical layout of the brain alone. However, the fact that the LSM can improve upon the predictive performance of this baseline indicates that there are additional principles at play, confirming recent work on generative principles of the connectome [57, 58].

There are a number of ways in which the link prediction approach may be extended in future work. First, following [59, 60], a nonparametric variant of the model may be constructed that learns the dimensionality $\hat{D}$ of the latent space from the data itself. This avoids the need for a cross-validation procedure, as all the data can be used to train the model and learn $\hat{D}$ simultaneously. Another extension could describe the FLNe weights in the macaque cerebral cortex data [45] as a continuous variable rather than the currently used thresholded categorization. We have refrained from this approach in order to present one model that was applicable to all three data sets, but it is to be expected that continuous weights better inform the latent distances than the ad-hoc ordinal representation of connection strengths, and subsequently increase prediction performance. A further model extension consists of adding other covariates of the regions to the model, such as cortical thickness. In [25], a general framework for such an approach is described.

A few studies are related to the presented work and have analyzed the spatial embeddedness of structural connectivity. For example, [43] use the 29 × 29 submatrix of fully observed connectivity in the macaque cerebral cortex to predict global graph-theoretical properties of the full 91 × 91 connectome. Here, it is assumed that the fully observed submatrix is representative of the entire connectome. This relates to our observation about the (relative) degree of regions in the data and in the predicted connectome, which have been found to be quite similar in the macaque cerebral cortex data. [61] consider a more abstract notion of topological dimensionality. Here, different nervous systems as well as computer circuits are studied and found to have a higher topological complexity than the physical embedding of the networks would suggest. As [61] argue, this implies that connectomes optimize for a trade-off between minimal wiring length and maximum topological complexity. Finally, [62] demonstrate that many large-scale features of connectivity in mouse, macaque and human can be explained by simple generative mechanisms based on spatial embedding.

We have demonstrated the usage of the latent space model for prediction of connectivity on animal tracer data. The advantage of tracer data over other modalities is their reliability (as, for example, dMRI-based structural connectivity estimates are often accompanied by uncertain estimates [63, 64]), which allowed us to evaluate the performance of link prediction using the latent space model. In terms of application however, other modalities may benefit more from our approach. For example, dMRI in combination with tractography is often used to estimate structural connectivity *in vivo*, making it applicable to human subjects. This approach has a number of well-known shortcomings that affect the resulting connectomes [3–5]. By using the LSM approach, connections that are difficult to estimate in living human subjects may be predicted from the connections that were more easily obtained. Furthermore, recent advancements in electron microscopy imaging have enabled connectivity analysis at the single-cell resolution [65, 66]. Here, link prediction may be used to complete the connectivity that has not yet been probed and at the same time to obtain insight in the possible spatial embedding of connectivity at this scale. For completely observed connectomes where link prediction is not directly relevant, the LSM may still be used to contrast the latent embedding with the anatomical organization of the connectome, e.g. for the recently published mouse meso-scale connectome [67].

With the increase in available connectivity data, proper statistical analyses of connectomes is paramount for furthering our understanding of the brain [66, 68]. There are two sides to the coin of these analyses. On the one hand, latent variable models such as the one employed here and elsewhere [35, 60, 69] allow for a succinct description of otherwise dauntingly large amounts of data. At the same time, models that fit sufficiently well to the data can be used to predict the status of hitherto unobserved connectivity. As successful prediction is testament to a useful underlying model, the results we have shown here corroborate that spatial embedding of a connectome provides a sensible representation of the data.

## Supporting information

S1 Appendix Supplementary material. (PDF)
