# Correction: The missing link: Predicting connectomes from noisy and partially observed tract tracing data

**DOI:** 10.1371/journal.pcbi.1005478

**Published:** 2017-04-11

**Authors:** Max Hinne, Annet Meijers, Rembrandt Bakker, Paul H. E. Tiesinga, Morten Mørup, Marcel A. J. van Gerven

In the Funding section, the grant number from the funder Lundbeck Foundation is listed incorrectly. The correct grant number is: R105-9813.
