# Supplementary material for: The missing link: Predicting connectomes from noisy and partially observed tract tracing data
Source: PLoS Comput Biol. 2017 Jan 31;13(1):e1005374. doi: 10.1371/journal.pcbi.1005374 (PMC5308841; doi:10.1371/journal.pcbi.1005374)
Supplement: S1 Appendix — (PDF) [file pcbi.1005374.s001.pdf]

The Missing Link: Predicting Connectomes from Noisy and Partially Observed Tract Tracing Data

Supplementary Material

Max Hinne[1,*], Annet Meijers[1], Rembrandt Bakker[2], Paul H. E. Tiesinga[1], Morten Mørup[3] and Marcel A. J. van Gerven[1]

[1]Radboud University, Donders Institute for Brain, Cognition and Behaviour, the Netherlands
[2]Institute of Neuroscience and Medicine (INM-6), Institute for Advanced Simulation (IAS-6) and JARA BRAIN Institute I, Jülich Research Centre, Germany
[3]Technical University of Denmark, DTU Compute, Denmark
[*]m.hinne@donders.ru.nl

1. Generative models

Latent space model. The formal description of the generative model for the latent space embedding with asymmetric effects is as follows:

$$
\begin{aligned}
\rho_\delta &\sim \mathrm{U}(0,\infty) \\
\rho_\varepsilon &\sim \mathrm{U}(0,\infty) \\
\delta_i \mid \rho_\delta &\sim \mathcal{N}(0,\rho_\delta^2) \\
\varepsilon_i \mid \rho_\delta &\sim \mathcal{N}(0,\rho_\varepsilon^2) \\
\rho_d &\sim \mathrm{U}(0,\infty) \\
z_{id} \mid \rho_d &\sim \mathcal{N}(0,\rho_d^2) && -1 \leq z_{id} \leq 1 \\
b_k &\sim \mathrm{U}(-\infty,\infty) && k \in \{1,\ldots,K-1\}, \quad b_{k-1} < b_k, \\
& && b_0 = -\infty, b_K = \infty \\
\sigma &\sim \mathrm{U}(0,1) \\
\Phi(i,j,k) &= \int_{-\infty}^{h(i,j,k)} \mathcal{N}(x \mid 0,1)\,\mathrm{d}x \\
f_{ijk} &= \Phi(i,j,k) - \Phi(i,j,k-1) \\
a_{ij} \mid \mathbf{f}_{ij} &\sim \mathrm{Categorical}(\mathbf{f}_{ij}) && i \neq j \;,
\end{aligned}
$$

with $h(i,j,k) = (b_k - \|\mathbf{z}_i - \mathbf{z}_j\|_2 + \delta_i + \varepsilon_j)/\sigma$. The symbol $\sim$ should be read as 'follows the distribution'. In this model, a_{ij} represents the categorical class of the connection between nodes i and j. As the latent space model considers only the relative distances between nodes, the positions $\mathbf{z}$ may be arbitrarily scaled, rotated and translated throughout the latent space. In order to have the posterior distribution be consistent across different samples, we constrain the positions to lie within the D-dimensional unit hypercube by requiring $-1 \leq z_{id} \leq 1$. This implies a maximum distance between any two nodes of $2\sqrt{D}$.

Extending the model to integrate both anterograde and retrograde tracing data is straightforward by incorporating the additional likelihood term

$$r_{ij} \mid \mathbf{f}_{ij} \sim \mathrm{Categorical}(\mathbf{f}_{ij})$$

into the model. Here, r_{ij} represents the retrograde connection while the original a_{ij} parameter represents the anterograde connection. Notably, both types of observations depend on the same latent distances. Note that in the Hamiltonian Monte Carlo framework (see below), improper priors such as $\mathrm{U}(0,\infty)$ are allowed as long as the resulting posterior remains proper [1].

Latent eigenmodel. In the latent eigenmodel, the distance $l_{ij} = \|\mathbf{z}_i - \mathbf{z}_j\|_2$ is replaced by $l_{ij} = -\mathbf{z}_i^T \mathbf{\Lambda} \mathbf{z}_j$, with $\mathbf{\Lambda}$ a diagonal matrix with elements λ_{ii} drawn from a standard Gaussian distribution [2]. Other hyperpriors and random effects are the same as in the LSM.

$$
\begin{aligned}
\rho_\delta &\sim \mathrm{U}(0, \infty) \\
\rho_\varepsilon &\sim \mathrm{U}(0, \infty) \\
\delta_i \mid \rho_\delta &\sim \mathcal{N}(0, \rho_\delta^2) \\
\varepsilon_i \mid \rho_\delta &\sim \mathcal{N}(0, \rho_\varepsilon^2) \\
\rho_d &\sim \mathrm{U}(0, \infty) \\
z_{id} \mid \rho_d &\sim \mathcal{N}(0, \rho_d^2) & -1 \le z_{id} \le 1 \\
\lambda_{ii} &\sim \mathcal{N}(0, 1) \\
b_k &\sim \mathrm{U}(-\infty, \infty) & k \in \{1, \ldots, K-1\}, \quad b_{k-1} < b_k, \\
& & b_0 = -\infty, b_K = \infty \\
\sigma &\sim \mathrm{U}(0, 1) \\
\Phi(i, j, k) &= \int_{-\infty}^{h(i,j,k)} \mathcal{N}(x \mid 0, 1)\, \mathrm{d}x \\
f_{ijk} &= \Phi(i, j, k) - \Phi(i, j, k-1) \\
a_{ij} \mid \mathbf{f}_{ij} &\sim \mathrm{Categorical}(\mathbf{f}_{ij}) & i \ne j \ ,
\end{aligned}
$$

with $h(i, j, k) = (b_k + \mathbf{z}_i^T \mathbf{\Lambda} \mathbf{z}_j + \delta_i + \varepsilon_j)/\sigma$.

Empirical class frequency baseline. In the first baseline, we assume the probability distribution of class weights $\mathbf{f}$ is shared across all connections. Furthermore, we place a flat prior on $\mathbf{f}$, e.g.:

$$
\begin{aligned}
\mathbf{f} &\sim \mathrm{Dirichlet}(\mathbf{1}_K) \\
a_{ij} \mid \mathbf{f} &\sim \mathrm{Categorical}(\mathbf{f}) \ ,
\end{aligned}
$$

so that the posterior can be obtained analytically as $P(\mathbf{A} \mid \mathbf{f}) = \prod_{i \ne j} \mathrm{Dirichlet}(\mathbf{1}_K + \boldsymbol{\alpha})$, with $\alpha_k = \sum_{i \ne j} \mathbb{1}[a_{ij} = k]$.

Random effects baseline. The random effects baseline assumes there is no latent space, but that connectivity is explained entirely by the random effects $\boldsymbol{\delta}$ and $\boldsymbol{\varepsilon}$:

$$
\begin{aligned}
\rho_\delta &\sim \mathrm{U}(0, \infty) \\
\rho_\varepsilon &\sim \mathrm{U}(0, \infty) \\
\delta_i \mid \rho_\delta &\sim \mathcal{N}(0, \rho_\delta^2) \\
\varepsilon_i \mid \rho_\delta &\sim \mathcal{N}(0, \rho_\varepsilon^2) \\
b_k &\sim \mathrm{U}(-\infty, \infty) & k \in \{1, \ldots, K-1\}, \quad b_{k-1} < b_k, \\
& & b_0 = -\infty, b_K = \infty \\
\sigma &\sim \mathrm{U}(0, 1) \\
\Phi(i, j, k) &= \int_{-\infty}^{h(i,j,k)} \mathcal{N}(x \mid 0, 1)\, \mathrm{d}x \\
f_{ijk} &= \Phi(i, j, k) - \Phi(i, j, k-1) \\
a_{ij} \mid \mathbf{f}_{ij} &\sim \mathrm{Categorical}(\mathbf{f}_{ij}) & i \ne j \ ,
\end{aligned}
$$

with $h(i, j, k) = (b_k + \delta_i + \varepsilon_j)/\sigma$.

Fixed-positions model. In the fixed-positions model, $\mathbf{z}$ is simply taken from the anatomical locations of the ROI. This leaves

$$\begin{aligned}
\rho_\delta &\sim \mathrm{U}(0,\infty) \\
\rho_\varepsilon &\sim \mathrm{U}(0,\infty) \\
\delta_i \mid \rho_\delta &\sim \mathcal{N}(0,\rho_\delta^2) \\
\varepsilon_i \mid \rho_\delta &\sim \mathcal{N}(0,\rho_\varepsilon^2) \\
b_k &\sim \mathrm{U}(-\infty,\infty) && k \in \{1,\ldots,K-1\}, \quad b_{k-1} < b_k, \\
& && b_0 = -\infty, b_K = \infty \\
\sigma &\sim \mathrm{U}(0,1) \\
\Phi(i,j,k) &= \int_{-\infty}^{h(i,j,k)} \mathcal{N}(x \mid 0,1)\,\mathrm{d}x \\
f_{ijk} &= \Phi(i,j,k) - \Phi(i,j,k-1) \\
a_{ij} \mid \mathbf{f}_{ij} &\sim \mathrm{Categorical}(\mathbf{f}_{ij}) && i \neq j \quad ,
\end{aligned}$$

with again $h(i,j,k) = (b_k - \|\mathbf{z}_i - \mathbf{z}_j\|_2 + \delta_i + \varepsilon_j)/\sigma$, but now $\mathbf{z}$ is observed.

2. Implementation

The models are implemented using the probabilistic programming language Stan [**?**] and MatlabStan [3], which interfaces Stan and Matlab. Stan implements the no-U-turn Hamiltonian Monte Carlo sampler [4]. For each different model, four parallel sampling chains are executed. Convergence to the posterior distribution is determined by computing the potential scale reduction factor (PSRF) [5] for parameters l_{ij}, $\mathbf{f}_{ij}$ and σ (where applicable). Once all PSRF scores are below 1.1 (typically after 6 000 − 10 000 iterations), the chains are considered to be converged[1]. Subsequently, the chains are merged and downsampled to 1 000 samples for efficient further analysis.

3. Computational demands

Per iteration of the Hamiltonian Monte Carlo algorithm, a total of $(D+2)p+K+2$ parameters need to be estimated. However, making general claims about the computational cost of the HMC approach is difficult as convergence depends the ease of which the latent positions can be determined, which in turn depends on the dimensionality D and the latent structure in the data. For example, we noticed that during the cross-validation procedure, the $D = 1$ case was easy to compute, but difficult to obtain convergence for (taking as much as 10 000 iterations), while higher dimensional latent spaces had more computational cost per iteration, yet converged much faster (in as few as 4 000 iterations). To provide a guideline for the efficiency of the approach, Fig. 1 shows the computation time per 100 iterations for the data used in this paper, using a single Intel Xeon CPU E5-2670 @ 2.60GHz per sampling chain. The approximately linear trends that are shown in these results may be used to extrapolate running times for connectomes with a larger number of nodes than used here. For example, prediction of connectivity for a connectome of 1 000 nodes, using a 2-dimensional latent space and the same hardware as above, should take roughly four days to compute.

4. Connectivity for $D = \{1, \ldots, 5\}$

Figures 2–6 show the predicted connectomes for all considered latent dimensionalities. The dimensionality with the optimal generalization performance is indicated. The figures show clearly how a one-dimensional latent space has difficulty capturing the structure in the data, and that increasingly higher dimensionalities fit increasingly better.

---

[1]The latent eigenmodel proved to have difficulty converging fully when using the data fusion approach for the mouse neocortex data, for dimensions $D \geq 3$. For these cases, we considered the model to be converged when at least 80% of the parameters had a PSRF score below 1.1.

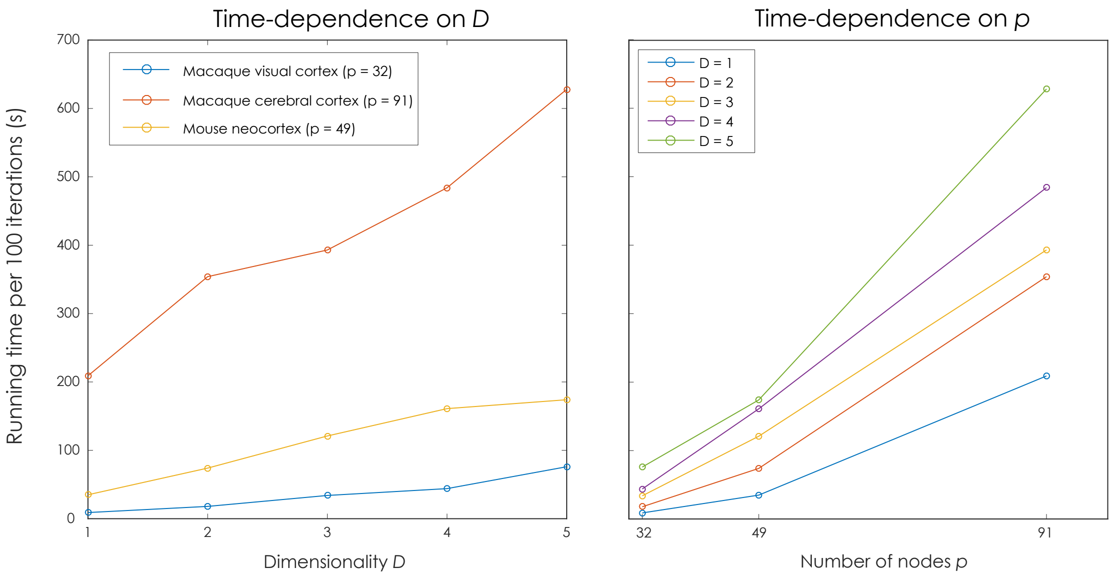

FIGURE 1. Running time of 100 iterations of the Hamiltonian Monte Carlo algorithm on each of the different data sets that are considered in the main text. The figures confirm the (approximately) linear dependence on the most important parameters of the model; the dimensionality D (left panel) and the number of nodes in the connectome p (right panel).

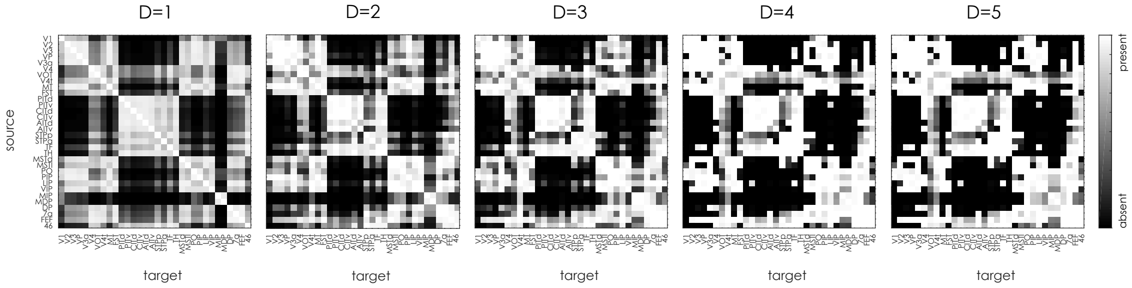

FIGURE 2. Predicted connectomes for $D = \{1, \ldots, 5\}$ for the macaque visual connectome.

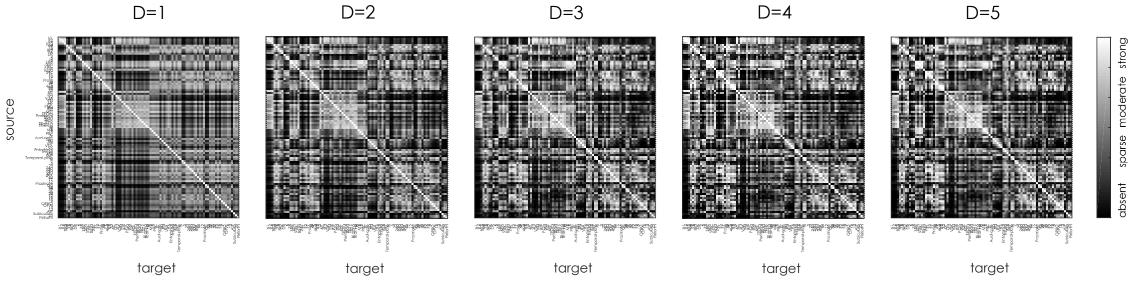

FIGURE 3. Predicted connectomes for $D = \{1, \ldots, 5\}$ for the macaque cerebral connectome.

5. PREDICTION PERFORMANCE FOR RECIPROCAL AND NON-RECIPROCAL CONNECTIONS

In the macaque visual cortex data, 540 connections are observed in both directions, while 113 connections are known only in one direction. For the macaque cerebral cortex, these numbers are

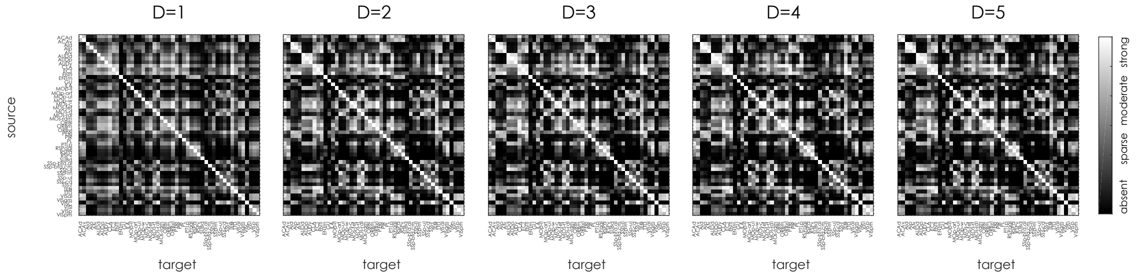

FIGURE 4. Predicted connectomes for $D = \{1, \ldots, 5\}$ for the mouse anterograde connectome.

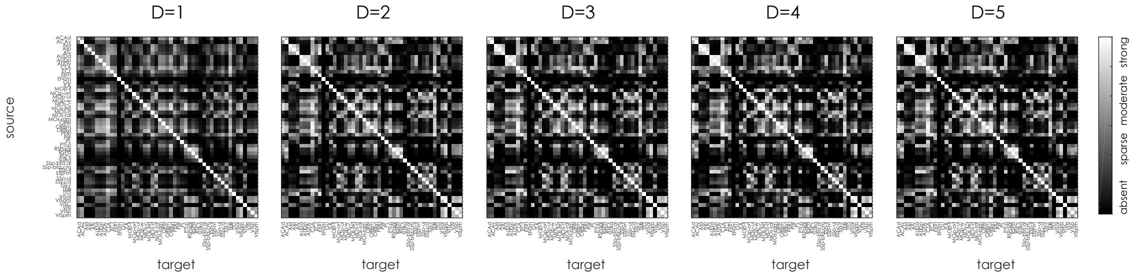

FIGURE 5. Predicted connectomes for $D = \{1, \ldots, 5\}$ for the mouse retrograde connectome.

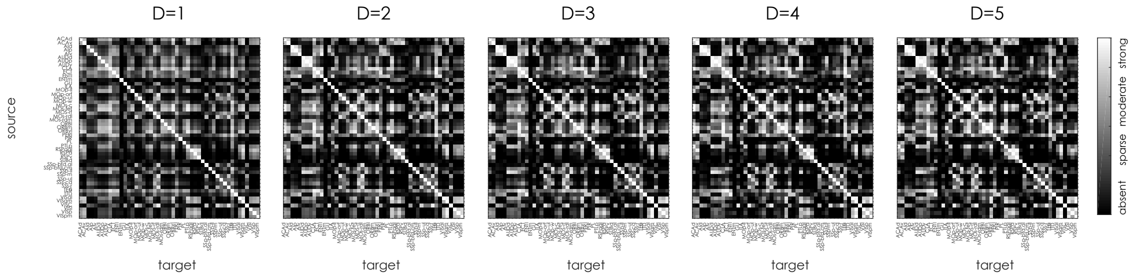

FIGURE 6. Predicted connectomes for $D = \{1, \ldots, 5\}$ for the mouse connectome using both anterograde and retrograde data.

812 and 1798, respectively[2]. To investigate whether knowing both the anterograde and retrograde observation for a potential connection affects the prediction performance, we computed the cross-validation performance measures for these different types of connections separately. Figure 7 shows these results. In general, there is no qualitative difference in the relative performances of the different methods and baselines when disentangling the reciprocal and non-reciprocal connections.

6. Changes in connectivity

Tables 1 and 2 show for the macaque visual system the change in relative degree when comparing the empirical connectome with the connectome completed by the LSM. Similarly, Tables 3 and 4 show for the macaque cerebral cortex the change in relative degree when comparing the empirical connectome with the connectome completed by the LSM.

[2]For the mouse neocortex data, all connections have been observed reciprocally, and this data set has hence been ignored in this analysis.

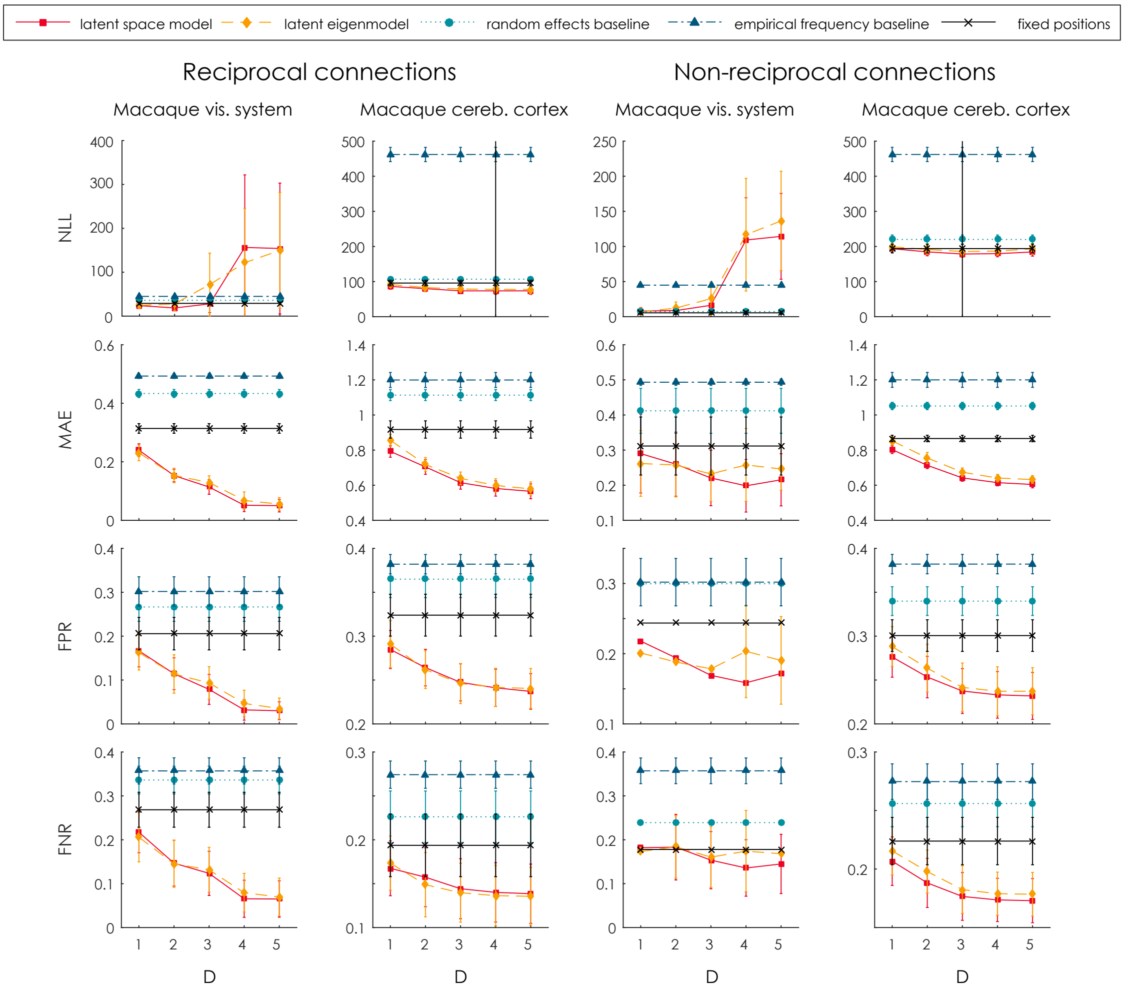

FIGURE 7. **Model performance.** The prediction performance of the latent space model, the latent eigenmodel and the baseline approaches, quantified using the negative log-likelihood (NLL), the mean absolute error (MAE), the false-positive rate (FPR) and the false-negative rate (FNR), for only reciprocal connections (left two columns) and only non-reciprocal connections (right two columns). All measures are obtained using ten-fold cross-validation. Error bars indicate one standard deviation over the ten folds.

REFERENCES

[1] Bob Carpenter, Andrew Gelman, Matthew Hoffman, Daniel Lee, Ben Goodrich, Michael Betancourt, Marcus Brubaker, Jiqiang Guo, Peter Li, and Allen Riddell. Stan: A probabilistic programming language. *Journal of Statistical Software*, 76(1):1–32, 2017.

[2] P D Hoff. Modeling homophily and stochastic equivalence in symmetric relational data. In J. C. Platt, D. Koller, Y. Singer, and S. T. Roweis, editors, *Advances in Neural Information Processing Systems 20*, pages 657–664. Curran Associates, Inc., 2008.

[3] B Lau. MatlabStan: the MATLAB interface to Stan, 2015. URL `http://mc-stan.org/matlab-stan.html`.

[4] M D Hoffman and A Gelman. The No-U-Turn sampler: Adaptively Setting Path Lengths in Hamiltonian Monte Carlo. *Journal of Machine Learning Research*, 15:30, 2014.

[5] A Gelman and D B Rubin. Inference from iterative simulation using multiple sequences. *Statistical Science*, 7(4):457–472, 1992.

Table 1. Relative observed and predicted degrees for each ROI in the macaque visual system connectome, for anterograde connections. The relative degree is defined as the number of connections divided by the number of possible connections. Rows are sorted by the difference in relative degree, in descending order.

ROI	**Observed**	**Predicted**	ROI	**Observed**	**Predicted**
V4t	0.89	0.61	STPa	0.26	0.32
FEF	0.92	0.72	MDP	0.11	0.17
PITv	0.21	0.40	V3a	0.46	0.52
AITd	0.24	0.38	MIP	0.11	0.17
VOT	0.71	0.59	V4	0.67	0.61
MSTl	0.42	0.54	7a	0.45	0.50
CITv	0.24	0.35	VIP	0.53	0.57
PITd	0.25	0.36	LIP	0.70	0.66
CITd	0.24	0.34	TF	0.59	0.62
DP	0.40	0.50	VP	0.45	0.48
TH	0.36	0.45	PIP	0.50	0.52
MSTd	0.70	0.61	FST	0.62	0.64
PO	0.39	0.47	V3	0.48	0.49
AITv	0.24	0.31	V1	0.26	0.27
STPp	0.36	0.43	V2	0.48	0.48
46	0.53	0.47	MT	0.53	0.53

Table 2. Relative observed and predicted degrees for each ROI in the macaque visual system connectome, for retrograde connections. The relative degree is defined as the number of connections divided by the number of possible connections. Rows are sorted by the difference in relative degree, in descending order.

ROI	**Observed**	**Predicted**	ROI	**Observed**	**Predicted**
CITd	0.06	0.33	V3a	0.44	0.52
PITd	0.13	0.35	TH	0.40	0.46
DP	0.75	0.55	V4	0.68	0.62
FEF	0.94	0.75	STPa	0.27	0.32
MIP	0.00	0.17	MSTl	0.50	0.55
MDP	0.00	0.16	AITd	0.33	0.37
V4t	0.75	0.59	V3	0.45	0.48
LIP	0.47	0.59	FST	0.68	0.65
VIP	0.43	0.54	PITv	0.39	0.42
46	0.65	0.54	7a	0.56	0.54
V2	0.38	0.48	STPp	0.44	0.46
PIP	0.37	0.47	AITv	0.29	0.31
VP	0.35	0.45	V1	0.26	0.27
CITv	0.26	0.34	MSTd	0.58	0.59
VOT	0.50	0.58	MT	0.50	0.51
PO	0.59	0.51	TF	0.63	0.63

Table 3. Relative observed and predicted degrees for each ROI in the macaque cerebral cortex connectome, for anterograde connections. The relative degree is defined as the number of connections divided by the number of possible connections. Rows are sorted by the difference in relative degree, in descending order.

ROI	**Observed**	**Predicted**	ROI	**Observed**	**Predicted**
12	1.76	1.798662	9	1.21	1.20
LIP	1.93	1.839565	MT	1.17	1.18
STPi	1.83	1.829971	F4	1.14	1.18
PGa	1.62	1.618966	V2	1.24	1.19
46v	1.62	1.641715	PBr	1.07	1.16
23	1.83	1.634263	TEOm	0.93	1.05
Insula	1.76	1.715188	LB	1.03	1.24
STPc	1.76	1.738232	24d	1.21	1.10
8B	1.56	1.533170	TEO	0.90	1.02
MST	1.72	1.595749	7op	1.10	1.02
9-46v	1.48	1.550750	S2	0.90	1.02
STPr	1.59	1.674020	29-30	1.07	1.04
Temporal-pole	1.34	1.529253	ProM	0.90	0.99
8m	1.59	1.584705	TPt	1.07	1.06
7A	1.69	1.581570	PBc	0.97	1.05
TH-TF	1.41	1.475802	31	1.03	0.95
46d	1.52	1.474743	DP	1.03	1.04
9-46d	1.52	1.463230	OPAI	0.62	0.89
TEam-a	1.45	1.532129	32	0.83	0.95
13	1.21	1.367773	F6	1.07	0.99
45B	1.28	1.360930	Pi	0.76	1.02
OPRO	1.14	1.279584	7B	0.97	0.94
F5	1.48	1.490845	2	0.97	1.02
24b	1.59	1.412569	PIP	0.79	0.84
Perirhinal	1.48	1.545821	V3	0.83	0.90
24a	1.21	1.323258	TEad	0.69	0.91
F7	1.45	1.356939	3	0.86	0.86
8l	1.31	1.385450	F1	1.14	0.96
FST	1.24	1.260681	14	0.69	0.89
8r	1.24	1.357227	Aud-core	0.66	0.90
MB	1.38	1.531420	V6A	0.79	0.84
24c	1.41	1.232569	V3A	0.69	0.72
45A	1.14	1.302214	AIP	0.59	0.63
TEav	1.14	1.300204	5	0.90	0.83
44	1.24	1.290662	V4t	0.55	0.65
IPa	1.21	1.289748	Gu	0.45	0.72
F2	1.45	1.299643	VIP	0.86	0.85
TEpd	1.10	1.239588	25	0.38	0.65
TEpv	1.10	1.257874	MIP	0.72	0.74
10	1.00	1.076933	V1	0.52	0.59
7m	1.21	1.140865	V6	0.34	0.48
TEam-p	1.17	1.283718	Piriform	0.17	0.54
Entorhinal	1.00	1.246137	Prostriate	0.45	0.54
V4	1.14	1.155382	Subiculum	0.24	0.60
F3	1.21	1.064390	1	0.41	0.48
11	0.97	1.163655			

Table 4. Relative observed and predicted degrees for each ROI in the macaque visual system connectome, for retrograde connections. The relative degree is defined as the number of connections divided by the number of possible connections. Rows are sorted by the difference in relative degree, in descending order.

ROI	**Observed**	**Predicted**	ROI	**Observed**	**Predicted**
46d	1.13	1.22	V4	0.77	0.79
STPi	0.99	1.06	8B	1.27	1.30
7B	1.07	1.13	ProM	0.79	0.81
8l	2.00	1.95	F7	1.41	1.43
7A	1.33	1.38	MT	1.15	1.17
STPc	1.43	1.47	DP	1.22	1.21
F1	0.60	0.65	10	1.35	1.34
TEpd	1.09	1.13	5	0.86	0.87
F5	1.24	1.27	STPr	1.43	1.44
7m	1.52	1.49	8m	1.70	1.71
F2	1.05	1.09	PBr	1.20	1.19
V2	0.68	0.71	V1	0.73	0.72
9-46v	1.64	1.66	24c	1.20	1.20
2	0.74	0.76	9-46d	1.62	1.62
TEO	1.18	1.20			
